# Emerging Concepts in Transesophageal Echocardiography

**DOI:** 10.12688/f1000research.7169.1

**Published:** 2016-03-14

**Authors:** Cory Maxwell, Ryan Konoske, Jonathan Mark

**Affiliations:** 1 Department of Anesthesiology, Veterans Affairs Medical Center, Durham, NC, USA; 2 Department of Anesthesiology, Duke University Medical Center, Durham, NC, USA

**Keywords:** transesophageal echocardiography, cardiac ultrasound, cardiac imaging, Three-dimensional imaging, Strain imaging, arrhythmias

## Abstract

Introduced in 1977, transesophageal echocardiography (TEE) offered imaging through a new acoustic window sitting directly behind the heart, allowing improved evaluation of many cardiac conditions. Shortly thereafter, TEE was applied to the intraoperative environment, as investigators quickly recognized that continuous cardiac evaluation and monitoring during surgery, particularly cardiac operations, were now possible. Among the many applications for perioperative TEE, this review will focus on four recent advances: three-dimensional TEE imaging, continuous TEE monitoring in the intensive care unit, strain imaging, and assessment of diastolic ventricular function.

## Introduction

**Figure 1.**
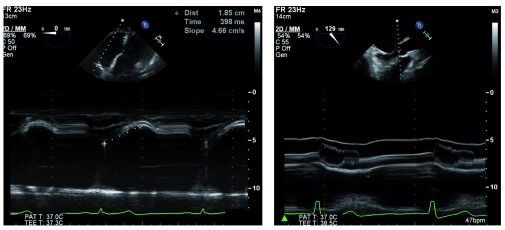
M-mode echocardiography is available on most modern transesophageal echocardiography probes. It is clinically useful for diagnosing and characterizing many clinical conditions, such as right ventricular function, by measuring tricuspid annular plane systolic excursion (left) or assessing dynamic left ventricular outflow tract obstruction hallmarked by fluttering and premature closure of the aortic valve leaflets (right). 2D, two-dimensional; bpm, beats per minute; MM, M-mode; PAT T, patient temperature; TEE T, transesophageal echocardiography temperature.

Clinical cardiac ultrasound, or echocardiography, is a technology that was pioneered in the post-World War II era with evolving naval sonar technology. Initial images consisted of the time-motion approach, displaying images along a single vector over time, or what we term today “M-mode” imaging ^1^ ( Figure 1). Over the subsequent 60 years, technological advances facilitated the introduction of two-dimensional (1971) ^2^ and three-dimensional (3D) (1990s) ^3– 5^ imaging into clinical practice and have led to greater accessibility and ease of interpretation by clinicians. Transesophageal echocardiography (TEE) was introduced in 1977 ^6^ and offered imaging through a new acoustic window sitting directly behind the heart, allowing improved evaluation of many cardiac conditions. It was only a short time until TEE was applied to the intraoperative environment (in 1981) ^7^, as investigators quickly recognized that continuous cardiac evaluation and monitoring during surgery, particularly cardiac operations, were now possible. Perioperative TEE guidelines were first established by the joint efforts of the American Society of Anesthesiologists and the Society of Cardiac Anesthesiologists in 1996 ^8^. European guidelines for this procedure soon followed, and there have been multiple updates published in recent years as newer TEE technologies have been developed ^9– 12^. The recent technological advances have significantly improved the perioperative care of patients with complex cardiac pathophysiology and will continue to advance both diagnostic and prognostic abilities of physicians via ultrasonography.

## Three-dimensional imaging

**Figure 2.**
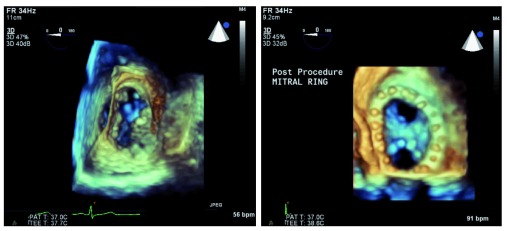
Three-dimensional assessment of the mitral valve has led to improved identification of pathology and guided repair. The native mitral valve (left) shows an anterior leaflet cleft and a posterior leaflet prolapse. Surgical repair includes mitral annuloplasty ring and Alfieri stitch (right), resulting in two distinct inflow orifices. 3D, three-dimensional; bpm, beats per minute; PAT T, patient temperature; TEE T, transesophageal echocardiography temperature.

**Figure 3.**
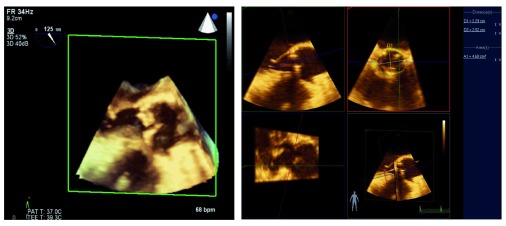
The three-dimensional view of the left ventricular outflow tract (left) allows precise measurement of the aortic valve annulus (right). This technique is used to guide percutaneous aortic valve replacement and has been shown to be equivalent to computed tomography angiography for this purpose. 3D, three-dimensional; bpm, beats per minute; PAT T, patient temperature; TEE T, transesophageal echocardiography temperature.

Most major manufacturers of TEE equipment and software have developed and improved 3D technology in recent years. Owing to the limitations of the physical properties of sound, tissue, and the TEE piezoelectric crystals, both the temporal resolution and the spatial resolution are significantly decreased in this imaging modality. Advances have improved real-time 3D imaging to the point where it has now become diagnostic-quality in multiple clinical scenarios, necessitating consensus clinical guidelines for utilization ^13^. For example, precise placement of percutaneous mitral valve edge-to-edge repair devices using 3D TEE alone has been described ^14^. This live imaging is accomplished without the use of ionizing radiation or contrast dye, both of which are potentially harmful. The temporal resolution of imaging can be improved by decreasing the visual field or sector width or by gated acquisition (dividing the imaging sector into two, four, or six sections and splicing these images back together). Though helpful for improved resolution, gated acquisition is more demanding from a clinical perspective because artifact-free images are impaired by patient movement (including cardiac translation during respiration), cardiac arrhythmias, and surgical electrocautery. As a result, higher-resolution imaging is generally limited to the intraoperative setting, when patients are anesthetized and mechanically ventilated, so that ventilation can be interrupted to provide a cardiac target that is not distorted by respiratory movements. The improved imaging has been particularly helpful in mitral valve repair ^15^ ( Figure 2) and percutaneous transcatheter aortic valve replacement ^16^ ( Figure 3).

In addition to advances in 3D image quality, improvements in post-processing capabilities have led to new applications. Several investigators have shown that 3D modeling of the mitral valve can assist with surgical decision-making and improve the quality of surgical repair or predict dynamic left ventricular outflow tract obstruction after the procedure ^17^. In patients undergoing transcatheter aortic valve replacement, computed tomographic angiography is the standard of care for measuring the aortic valve annulus; precise measurement is important to prevent an oversized valve and damage to the aorta or an undersized valve leading to perivalvular leaks. Accuracy of 3D TEE measurement of the aortic valve annulus has been shown to be equivalent to that of computed tomographic angiography and can be done without exposure to radiation or contrast dye during the actual procedure ^18, 19^ ( Video 1).

Video 1 Multiple applications of three-dimensional (3D) echocardiography are demonstrated, including precise sizing of the aortic annulus for transcatheter aortic valve replacement (TAVR), evaluation of mitral valve repair, confirmation of left ventricular assist device (LVAD) inflow cannula position, and real-time guidance of wire and catheter insertion for percutaneous procedures such as mitral valvuloplasty. bpm, beats per minute; LA, left atrium; LV, left ventricle; LVOT, left ventricular outflow tract; PAT T, patient temperature; RV, right ventricle; TEE T, transesophageal echocardiography temperature. Click here for additional data file. Copyright: © 2016 Maxwell C et al. 2016 Data associated with the article are available under the terms of the Creative Commons Zero "No rights reserved" data waiver (CC0 1.0 Public domain dedication).

## Continuous critical care monitoring

The volume status and cardiac function of critically ill patients, particularly following cardiac surgery, have traditionally been determined by using a pulmonary artery catheter, which is invasive and carries a small but non-zero risk of pulmonary artery rupture and other life-threatening complications. The efficacy of TEE as an alternative modality to accurately differentiate between cardiogenic shock or hypovolemic shock and guide management in the intensive care unit (ICU) is well established ^20^. An emerging technology is that of the “continuous TEE” using a disposable TEE probe, which can remain in the patient for up to 72 hours in the ICU. This has shown some promise as a useful diagnostic tool for tracheally intubated patients with complex pathophysiology. The use of continuous TEE has been demonstrated to help predict neurologic outcomes of patients following cardiac arrest which is treated with hypothermic cooling ^21^. Additionally, following valve replacement, continuous TEE monitoring has been helpful in guiding management in patients with hemodynamic instability ^22^. Continuous TEE monitoring is a fledgling technology and as such does not have extensive cost efficacy or outcome data associated with it. Nonetheless, its role in the management of critically ill patients may be expanded and should be better defined in coming years ( Video 2).

Video 2 Evaluation of hypotension in the intensive care unit (ICU) is aided by transesophageal echocardiography (TEE) to establish volume status, cardiac function, and the presence or absence of hemodynamically significant valvular stenosis or regurgitation. 2D, two-dimensional; BPM, beats per minute; LA, left atrium; LV, left ventricle; Pat. T, patient temperature; RV, right ventricle; TEE T, transesophageal echocardiography temperature. Click here for additional data file. Copyright: © 2016 Maxwell C et al. 2016 Data associated with the article are available under the terms of the Creative Commons Zero "No rights reserved" data waiver (CC0 1.0 Public domain dedication).

## Strain imaging

One of the more recent, yet infrequently used, technologies in clinical practice is myocardial strain analysis. Strain (ε), defined as (L − L _0_)/L _0_ where L = length at end-systole and L _0_ is the initial length at end-diastole, is a unit-less percentage change in myocardial deformation. Directly tracking myocardial compression and expansion throughout the cardiac cycle may offer a more load-independent measure of cardiac function. Strain essentially measures changes in sarcomere length over each cardiac cycle as opposed to percentage volume change in the left ventricular cavity, which is highly susceptible to loading, chronotropic, and inotropic states. This is particularly pertinent during intraoperative imaging, where there are abrupt changes in the aforementioned variables. Strain rate, or strain over time, may offer an even more load-independent measure of intrinsic myocardial function ^23^.

**Figure 4.**
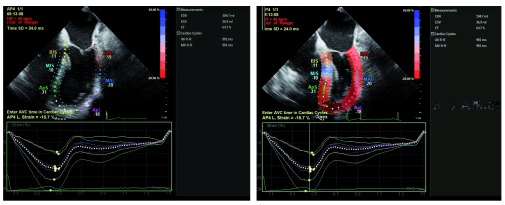
Post-processing of the two-dimensional echocardiographic images with strain software allows assessment of the motion and strain of the heart throughout the cardiac cycle (left, diastole) (right, systole). ApL, apical lateral; ApS, apical septal; BAL, basal anterolateral; BIS, basal inferoseptal; bpm, beats per minute; HR, heart rate; MAL, mid anterolateral; MIS, mid inferoseptal; SD, standard deviation; AVC, aortic valve closure; AVR-R, aortic valve R-R interval; EDV, end-diastolic volume; EF, ejection fraction; ESV, end-systolic volume; MVR-R, mitral valve R-R interval.

Two modalities have been developed to assess ventricular deformation or strain. Tissue Doppler imaging uses relative tissue velocities but suffers from the limitations of Doppler imaging, most notably angle dependence ^24^. The second method, speckle-tracking echocardiography, measures strain with acoustic markers or “speckles” on B-mode imaging and tracks their motion relative to one another. Although this method requires an adequate frame rate, it has emerged as a more robust method of strain measurement because speckles can be tracked at any angle. Longitudinal, circumferential, and radial strain can all be analyzed fully with this method. With multiple options for imaging platforms and software, strain is rapidly becoming a viable tool with multiple applications for the echocardiographer ^25^ ( Figure 4).

Most of the research for use of strain in the clinical arena is focused on patients with diagnosed heart failure and preserved left ventricular ejection fraction, often termed diastolic heart failure. Patients who have normal left ventricular ejection fraction but who demonstrate reduced global longitudinal strain values have more hospital readmissions for heart failure and suffer higher mortality ^26^. Similarly, in patients with chronic atrial fibrillation, reduced global longitudinal strain has been shown to be a stronger predictor than ejection fraction for adverse cardiac events ^27^. Echocardiographic strain analysis is also used to assess patients being considered for cardiac resynchronization therapy. Also known as bi-ventricular pacing, resynchronization therapy is aimed at correcting intraventricular electromechanical dyssynchrony and is reserved for patients with heart failure symptoms that meet certain criteria, such as QRS duration of more than 120 ms, left ventricular ejection fraction of less than 35%, and sinus rhythm ^28^. Recent investigations have focused on speckle-tracking echocardiography to characterize mechanical dyssynchrony as well as to optimize pacing lead positioning to achieve maximal ventricular synchrony ^29, 30^. Beyond its clinical applications in patients with heart failure, there is limited research evaluating the intraoperative application of speckle-tracking strain analysis, but a recent study has demonstrated an association of reduced global longitudinal strain with development of post-operative atrial fibrillation in patients undergoing isolated aortic valve replacement ^31^ ( Video 3).

Video 3 Speckle-tracking and strain analysis of the left ventricle delineates each ventricular segment and allows identification of local areas of variability in global longitudinal strain. ApL, apical lateral; ApS, apical septal; BAL, basal anterolateral; BIS, basal inferoseptal; bpm, beats per minute; HR, heart rate; MAL, mid anterolateral; MIS, medium inferoseptal; SD, standard deviation; AVC, aortic valve closure; AVR-R, aortic valve R-R interval; EDV, end-diastolic volume; EF, ejection fraction; ESV, end-systolic volume; MVR-R, mitral valve R-R interval. Click here for additional data file. Copyright: © 2016 Maxwell C et al. 2016 Data associated with the article are available under the terms of the Creative Commons Zero "No rights reserved" data waiver (CC0 1.0 Public domain dedication).

## Assessment of diastolic function

Diastology, or the study of cardiac diastolic function, has gained steam in the past decade. Historically, echocardiographic attention has been focused on the quantification of left ventricular systolic function. However, systolic dysfunction is not always the cause of heart failure: roughly 50% of patients with symptomatic heart failure have a preserved left ventricular ejection fraction ^32^. Furthermore, specific therapies for diastolic heart failure are limited. Current guidelines promulgated by the American College of Cardiology and the American Heart Association recommend blood pressure control and diuretics in volume overloaded states, but note that there is little evidence to support additional therapies ^33^.

Comprehensive assessment of left ventricular diastolic function has included multiple quantitative echocardiographic measurements, including pulsed wave Doppler assessment of mitral and pulmonary venous inflow velocities, tissue Doppler assessment of mitral annular velocities, color M-mode assessment of mitral inflow propagation velocity, and a variety of other methods. Many studies have looked at different measurement variables in the quantification of diastolic function. Therefore, in 2009, the European Association of Echocardiography, in conjunction with the American Society of Echocardiography, published guidelines for the assessment, quantification, and grading of diastolic function ^34^. With limited therapies available for diastolic heart failure, classification of diastolic dysfunction carries mostly a prognostic value rather than serving to guide treatment options. Similarly, intraoperative decision-making based on diastolic function classification in patients undergoing cardiac surgery has limited evidence to guide specific therapy.

**Figure 5.**
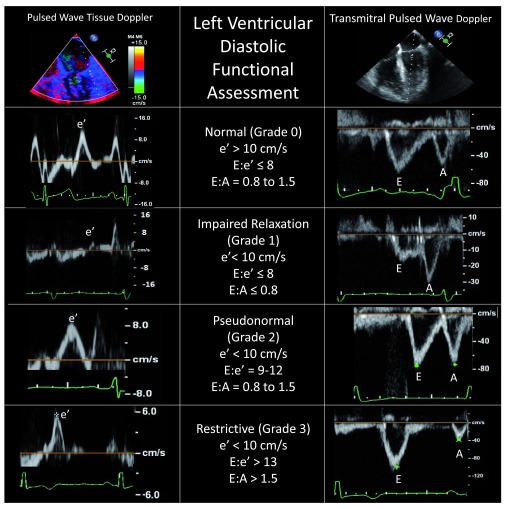
Assessment and classification of left ventricular diastolic function using pulsed wave tissue Doppler of the mitral annulus (left panels) and pulsed wave Doppler of the mitral inflow (right panels). Accurate identification of each Doppler spectral peak requires an accompanying electrocardiographic tracing. Conditions such as atrial fibrillation, mitral annular calcification, mitral valve surgery, or extracorporeal circulatory support generally preclude using these Doppler techniques. A, late mitral inflow velocity resulting from atrial contraction; E, early mitral inflow velocity; e′, early mitral annular velocity recorded from the lateral mitral annulus.

In 2011, Swaminathan *et al.* proposed a relatively simple and effective algorithm for intraoperative TEE evaluation and classification of left ventricular diastolic function ^35^. This algorithm incorporates the measure of mitral annular tissue velocity recorded from the lateral mitral annulus in early diastole (e′), with a value of more than 10 cm/s indicating normal diastolic function. Further stratification of left ventricular diastolic dysfunction for mitral tissue Doppler velocities of less than 10 cm/s uses the ratio of early mitral inflow velocities (E) to e′. Higher E/e′ ratios are indicative of worsening left ventricular diastolic function, as early mitral inflow velocities increase while annular velocities are reduced with later stages of diastolic dysfunction ( Figure 5). Despite the algorithm’s ease of use, there is an ongoing debate as to the clinical applicability of classifying degrees of left ventricular diastolic dysfunction. Although patients undergoing coronary artery bypass grafting who have left ventricular diastolic dysfunction are known to have an increased incidence of major adverse cardiac events ^35^, there are few targeted therapies for this condition and even fewer specific guidelines for perioperative management.

Proponents of intraoperative classification argue that hemodynamic manipulation based on the underlying class of diastolic dysfunction may improve stability in the perioperative period. For example, in patients with grade 1 dysfunction (e′ < 10 cm/s and E/e′ ≤ 8), also known as impaired relaxation, maintenance of sinus rhythm with an adequate diastolic filling time (slower heart rate) is favorable, owing to a longer time period for ventricular relaxation ( Figure 5). Conversely, patients presenting with more advanced (grade 2 or 3) left ventricular diastolic dysfunction (E/e′ ≥ 9) should be carefully monitored for increased intravascular volume because these patients typically have increased left atrial pressures and are at risk for development of pulmonary edema. Furthermore, higher heart rates will likely maintain cardiac output when stroke volume is relatively fixed, as seen in more advanced diastolic dysfunction ^36^.

Those who criticize efforts to assess and classify left ventricular diastolic function cite the limited evidence for targeted therapies ^37– 39^, including hemodynamic manipulation, in improving outcomes in these patients. Given the conflicting evidence for the effects of inotropic drugs on diastolic function, it is understandable that many clinicians remain skeptical of current efforts to use TEE to assess diastolic function parameters in the perioperative environment.

## Conclusions

With the pace of improvement in TEE technologies that we have seen since its introduction, it is likely that further refinements and novel imaging modalities will continue to be introduced in coming years. Three-dimensional echocardiography has made the diagnosis of complex pathologies more accurate and is essential for guiding procedural interventions in real time. Improvements in real-time 3D resolution will facilitate high-quality imaging without the artifacts caused by arrhythmias and cardiac translation. Continuous TEE monitoring in the ICU has shown some early promise and in coming years may replace the pulmonary artery catheter for hemodynamic evaluation of unstable tracheally intubated patients.

Speckle-tracking and strain analysis have now been incorporated into the management of patients with chronic heart failure, but the practical use of these methods in the perioperative setting has yet to be elucidated. Current challenges include the time-consuming nature of speckle-tracking methods, which are not conducive to measurement in a busy intraoperative setting, and the clinical utility of detecting subtle reductions in systolic function. There is some evidence that in patients with preserved ejection fractions, global longitudinal strain may have predictive value in the development of post-operative atrial fibrillation. Additional studies are needed to determine the relevance of these findings.

Although assessment and classification of diastolic function have improved and offer valuable prognostic information, there is currently little evidence to suggest that this leads to interventions that improve clinical outcomes. Despite the many advances in perioperative TEE in the past few decades, we still have opportunities ahead to improve the use of this technology for our patients.

## Data availability

The data referenced by this article are under copyright with the following copyright statement: Copyright: © 2016 Maxwell C et al.

Data associated with the article are available under the terms of the Creative Commons Zero "No rights reserved" data waiver (CC0 1.0 Public domain dedication). 

F1000Research: Dataset 1. **Video 1**, 10.5256/f1000research.7169.d115458 ^40^

F1000Research: Dataset 2. **Video 2**, 10.5256/f1000research.7169.d115459 ^41^

F1000Research: Dataset 3. **Video 3**, 10.5256/f1000research.7169.d115460 ^42^
